# How Do Perceived Social Support and Community Social Network Alleviate Psychological Distress During COVID-19 Lockdown? The Mediating Role of Residents' Epidemic Prevention Capability

**DOI:** 10.3389/fpubh.2022.763490

**Published:** 2022-04-18

**Authors:** Xu Zhao, Aolan Jin, Bin Hu

**Affiliations:** ^1^College of Economics and Management, China Three Gorges University, Yichang, China; ^2^School of Management, Huazhong University of Science and Technology, Wuhan, China

**Keywords:** COVID-19, community social network, perceived social support, psychological distress, epidemic prevention capability, public health emergency

## Abstract

**Aim:**

Following the outbreak of the COVID-19 epidemic, China adopted community isolation management measures. During the “lockdown” period, urban communities were the most basic prevention and control unit for the epidemic. The effectiveness of community epidemic prevention directly affects the spread of the virus and social stability. Therefore, the aim of this study was to explore the status quo and influencing factors of psychological distress.

**Methods:**

For this study, 1,430 community households were randomly selected in key cities affected by the epidemic, and a questionnaire survey was administered during the lockdown period. A structural equation model was used to analyse the influencing factors of community epidemic prevention effects. A total of 1,326 valid questionnaires were collected, with a valid response rate of 92.73%.

**Results:**

In this study, the differences in psychological distress among different community types were statistically significant (t = 58.41, *P* < 0.01). The results showed that epidemic prevention capability played a mediating role. The results of the high-order structural equation model analysis showed that perceived social support (β = −0.275, *P* = 0.000) and community social network (β = −0.296, *P* < 0.01) were significantly negatively correlated with psychological distress.

**Conclusions:**

Community social support indirectly relieves psychological anxiety and improves the effect of epidemic prevention by enhancing residents' ability to prevent epidemics. The community social network help residents reduce the risk of outbreaks and indirectly alleviate psychological distress.

## Introduction

The coronavirus disease (COVID-19) evoked a global pandemic in the spring of 2020. To effectively curb the spread of the disease, China implemented closed community management, which keeps people at a social distance (1). Twenty days after Wuhan was sealed off, the number of new suspected cases decreased by 53.5% compared with the peak period. However, the new lifestyle with limited living space and the unknown duration of the epidemic caused negative feelings, such as panic and anxiety among people (2, 3).

The key question is how to rely on two levels, the community and individuals, to quickly improve the epidemic response capability of residents in closed living spaces to create a peaceful and positive public epidemic prevention mentality (4). Community organizations provide scientific guidance and strict organization to offer public services and social support for residents (5). As a grassroots organization, the community timely begins the emergency management program, deploys the prevention and control plan, and takes emergency measures. In addition, the community actively deploys manpower and provides resources to protect people's basic livelihood. Voluntary organizations provide targeted services and care for disadvantaged groups. At the same time, they provide residents with emotional support, such as psychological assistance services, to reduce their psychological pressure and anxiety (6).

At the individual level, relying on the relationship capital accumulated by the family in the community as a non-institutional force, it forms an important auxiliary support. In the “unusual” situation of closed management, the residents in the community are more closely connected, and the community relationship network improves the residents' response capabilities in three ways: prevention and preparedness, crisis response capabilities, and recovery and reconstruction capabilities (7). On the one hand, trust and recognition among residents in the community affect the individual's judgment on the development trend of an incident, which is conducive to improving the family's preventive ability before an outbreak (8). On the other hand, the interactions in the community network can enhance the availability of residents' medical information and resources, thereby improving the health of residents. At the same time, social capital enriches people's professional prevention knowledge to reduce the impact of epidemic risk (9).

Although the social support of the community has a general gain effect, there are differences in organization forms and coordination mechanisms among different types of communities, such as commercial housing communities, transitional succession communities, traditional communities, unit communities, and suburban communities. In addition, the epidemic defense capability of community residents can also help them adjust their mentality in a timely manner to rationally and actively deal with the outbreak of COVID-19. First, the ability of prevention preparation enables residents to have a certain understanding of the epidemic situation and eliminate the fear of new infectious diseases. Moreover, the improvement of residents' crisis management ability can alleviate the anxiety caused by disease risk. Thirdly, recovery and reconstruction can effectively promote residents to return to work and production after the epidemic and realize the sustainable development of their livelihoods.

In summary, as the smallest unit of urban governance, the community is the basis and a key link for the prevention and control of public health emergencies. Therefore, to consolidate the effects of urban prevention and control in the post-epidemic era, it is necessary to study the mechanisms of community social networks and social support concerning residents' epidemic defense capability and psychological distress.

## Study Design

### Psychological Distress

In the period of urban closed management, coupled with the spread of the virus, the fear of unknown new infectious diseases makes people feel more anxious and helpless (10). The public mentality has social and group attributes, such as the individual's own situation, and health literacy directly affects mentality. However, the anxiety caused by the social panic caused by the epidemic needs to be transformed into a mature and healthy attitude to address it. In microblog text data from the epidemic period, it is obvious that the distortion of the epidemic network information strengthened the sensitivity of urban residents to social risks (11). In particular, excessive public attention to epidemic information will deepen the risk of transmission of group psychological distress (12). In view of the psychological distress of different subjects during the epidemic period, studies have mostly focused on measuring psychological stress states and attitudes. For example, psychological perception, self-rated health, subjective well-being, and other dimensions measured psychological distress (13). As the rapid spread of the epidemic creates a psychological burden on the populace, resident job categories and experiences can also have a differential impact (14). Therefore, large-scale community isolation mainly threatens the mental health status of residents, and alleviating psychological distress is an important puzzle to cope with the lockdown period. Therefore, psychological distress was the dependent variable in this study.

### Perceived Social Support and Community Social Network

Perceived social support is a way for residents to reduce psychological stress, relieve tension, and improve social resilience through social contacts, mainly referring to mental and physical help from family members, colleagues, and the community (15, 16). Current mechanistic models of the role of social support on mental health include the main effect model, buffer effect model, and dynamic effect model (17). Social support enables residents to correctly assess the severity of outbreaks, thereby improving people's mental health.

Since the community is the life carrier of residents with a common emotional and psychological identity, community support has become the first line of defense against disasters (18). Community health workers can be effectively engaged to provide psychosocial support at the community level (19). As the basic building blocks of urban governance, communities have the unique advantages of extensive mobilization and resource integration, dominating, and providing “quasi-formal support,” including subjective and objective support. Personal support refers to the emotional support perceived by residents and includes the extent of respected and understood experiences in the community. Objective support refers to the material support and services received. In response to the public crisis, the above support can promote the resilience of residents' behavioral and cognitive dimensions in stressful conditions by stabilizing their emotions. Social support under health shock also allows residents to perceive the help provided by others or organizations and alleviates individuals' emotional and physiological responses generated by health stress.

Social capital attached to social networks as a class of micro individual embeddedness resources, the functions can be summarized in four categories: information acquisition, reciprocal cooperation, structural support, and resource acquisition (20). Many studies have been carried out from the two perspectives of farmers' households and enterprises. But in recent years, the health effects involving informal community organizations have gradually become a focus, and their roles are reflected as follows: sharing family risks, promoting personal health, and information resource acquisition (21, 22). Therefore, the role of social networks in resource acquisition, risk response, and recovery and reconstruction may positively impact residents' defense against outbreaks (23, 24). The strong intergroup relationship of social networks enables social capital to override family accumulation in the community and generates obligations and identities to the community. Among these characteristics is cognitive embeddedness in community networks, and interactive learning identification clearly impacts health (25). Increased relationship density and connection will foster trust mechanisms, so having some stable community relationships can improve responsiveness during an outbreak (26). Together with individuals' ability to mobilize formal vs. informal resources from their own network to withstand the effects of disasters to restore normal life, the embeddedness of the community network is positively related to Epidemic defense capability.

### Epidemic Defense Capability

The ability of residents to deal with public health emergencies is mostly from two perspectives. One is the cognitive understanding of the epidemic. Residents' perceptions and attitudes toward disease influence their coping abilities and strategy effectiveness (27). For example, the COVID-19 transmission rate in Zhejiang residents is relatively low. However, the daily protection awareness of the people in Guangdong Province needs to be improved, and the ability to recognize the epidemic situation and distinguish rumors from facts is weak (28). The second is protection behavior. Compared with rural areas, the implementation rate of protection measures of urban residents is high, but there is still room for improvement (29). At present, the efficiency of protective measures is often used to measure the epidemic defense capability of residents, but the epidemic prevention capability mainly includes prevention, rapid response, self-help, recovery and reconstruction abilities (30). Therefore, this study measured the response level of public health events throughout the three stages before, during, and after the epidemic, including the awareness of the event and the protection ability of community residents. The positive coping style can reduce the residents' nervous reactions and relieve the public's panic in the face of public health emergencies (13). Through the investigation of the COVID-19 outbreak, we found that residents' attitudes (anxiety level and epidemic confidence) were significantly correlated with the behavioral dimension (protective measures and information acquisition) (31). In general, epidemic defense capabilities can effectively reduce psychological trauma and stress. Therefore, the epidemic defense capability can play a mediating role.

### Community Type

With China's social transformation, the functions of urban communities have gradually diversified, and the differences between community attributes are prominent. The social capital stock is heterogeneous in different types of communities. The subjective well-being of residents in high social capital communities is generally higher, which leads to differences in residents' emotions and health. At the same time, the higher the individual's trust in other community members, the better their perception of their health (32). Residents living in urban and rural communities are exposed to different risks (33, 34). A study of societies in Nanjing, China, showed that communities with a new construction age and a good built environment generally had lower levels of risk exposure. Those with higher education and social capital levels have lower sensitivity (35). Residents in affordable housing have weak community attachments and desire to move (36). Different community resources and service capabilities will enhance or reduce residents' perceived social support and affect epidemic defense capability. Therefore, community type acts as a moderator variable in the structural model of this study.

### Theoretical Foundation and Hypothesis Development

Most of the existing studies are limited to a single level of the social network or social support, and the effect of the interaction between the two on psychological stress has not been involved. Therefore, this article constructs a high-order factor structural equation model to analyze how the individual and community pathways alleviate psychological stress. At the same time, the mediating role of this mechanism was determined.

Social network theory states that communities are suitable places for weak ties (37) that “weak ties” are conducive to the flow of differentiated resources and information in community social networks, expanding resource transmission channels (38). On the one hand, social networks act on residents' medical resource acquisition and health care behaviors, exerting a significant positive influence on the level of health (39). On the other hand, after the SARS event, rural household medical security and the subsequent livelihood recovery of farmers' households were critically dependent on their own social network structure and function (40). Therefore, the community relationship acted as an informal support vector and played an active role in raising visit funds and improving psychological status. The ability of residents to benefit from social capital depends on the characteristics of network members and their close ties (41). The nature of social networks includes the scale, closeness, heterogeneity and reciprocity of the residents' network. Some scholars define community social networks from four dimensions: online learning, online trust, online interaction, and online reciprocity (42). Therefore, the community social networks in this study are divided into three levels: the heterogeneity of the relationship scale, the closeness of trust and mutual benefit, and the identity of interactive learning.

Social support generally refers to the general term for the behavior of certain social groups to use material and spiritual means to provide free assistance to vulnerable groups (43). Therefore, community social support among residents during the closed management period of the epidemic might have acted as a buffer to reduce resident stress. It significantly reduces negative emotional harm by alleviating physiological and psychological stress responses (44, 45). In public health emergencies, community social support provides emotional and instrumental help to relieve individual stress, and residents' perceptions are mainly focused on actual action and psychological state (46). Heterogeneous individuals also have different needs and use. Therefore, perceived social support can be divided into three aspects: subjective support, objective support, and support utilization (47). In addition, the type of community may also differ significantly, which will have a moderating effect between perceived social support and epidemic prevention capability.

**Figure 1:**
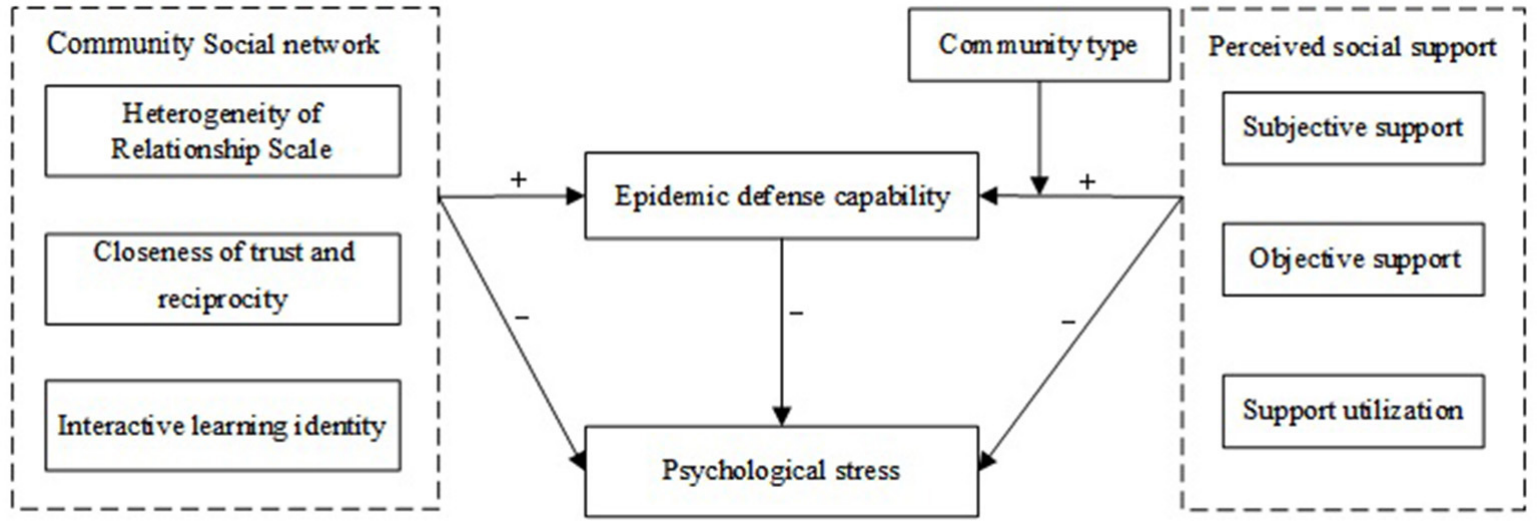
Structural equation model diagram.

According to the social network theory and social support theory reviewed above, the structural equation model was established in this study, depicted in Figure 1. The hypotheses of this study are as follows:

H1: Community social network has a positive impact on epidemic prevention capability.
H2: Community social network has a negative effect on psychological stress.
H3: Perceived social support has a positive effect on epidemic prevention capability.
H4: Perceived social support has a negative effect on psychological stress.
H5: Epidemic prevention capability has a negative effect on psychological stress.
H6: Epidemic prevention capability has a mediating effect on the relationship between perceived social support and psychological stress.
H7: Epidemic prevention capability has a mediating effect on the relationship between community social networks and psychological stress.
H8: Community type has a moderating effect on the relationship between perceived social support and epidemic prevention capability.

## Methodology and Measurement

### Data Collection and Sample

The survey was conducted in February 2020, when the communities were closed. Random sampling methods were used to administer online surveys to community households. The scope involved different types of communities in the key epidemic areas in Hubei Province and surrounding provinces.

**Figure 2:**
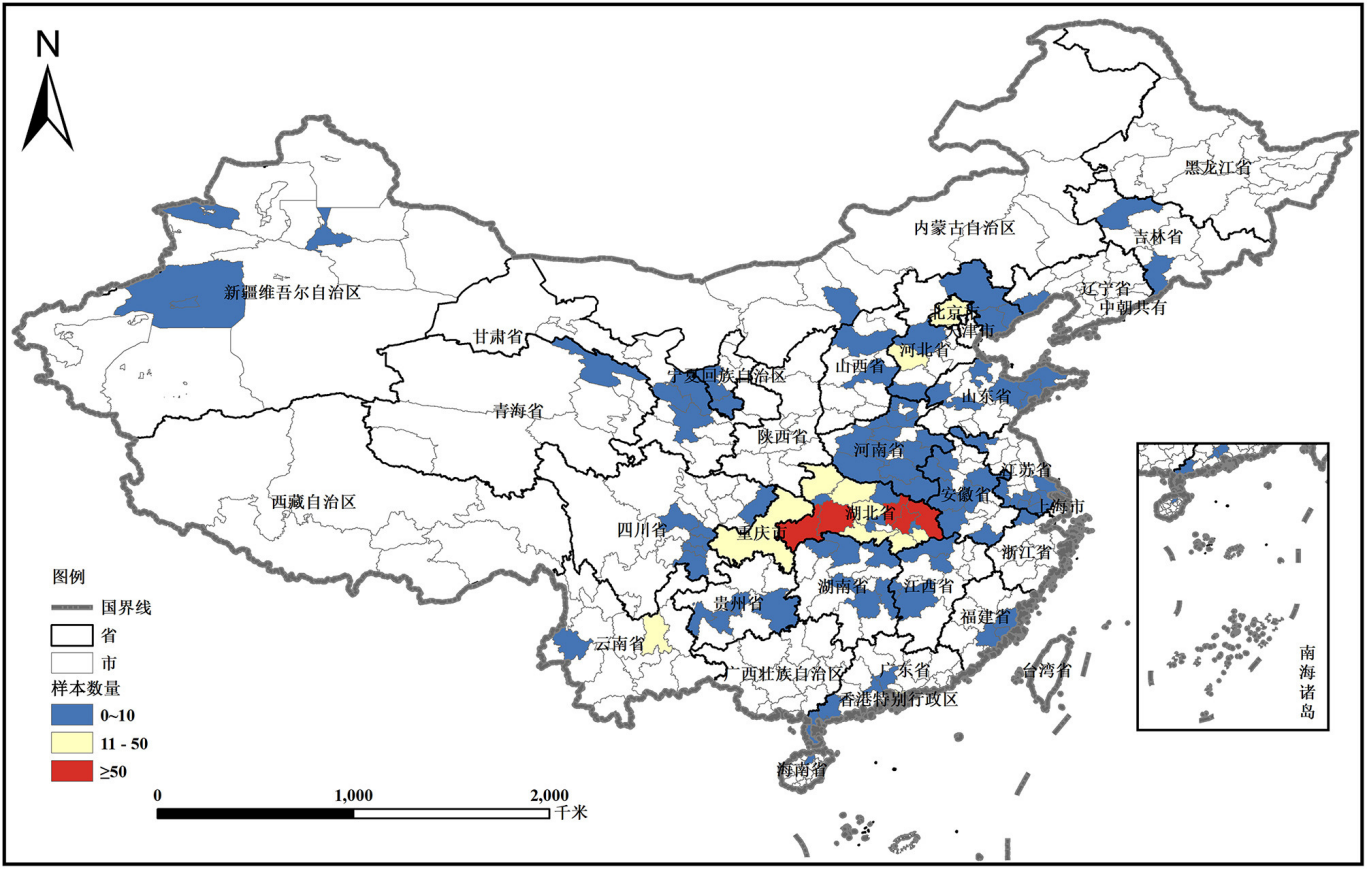
The location of the surveyed areas and sample distribution.

The sample consisted of 1,326 individuals, including 840 women and 486 men (see Table 1). The proportion in Hubei Province is 74.84%, and the rest are in Henan, Anhui, and Sichuan. Hubei Province was the site of the earliest outbreak of COVID-19 in China and the focus area of the epidemic in China, with 95% of the total number of cases and deaths in China at the beginning of the outbreak. In the Hubei sample, the proportion in the Wuhan City Circle is 65.98% (see Figure 2). Among the household heads, 91.41% were under 60 years old, and 73.53% had a bachelor's degree or above. This shows that most of the sample households are in the core area of the epidemic, and at the same time, they had sufficient awareness of their own situations and anti-epidemic statuses.

**Table 1:** Descriptive characteristics of the sample.

**Statistical category**	**Sample characteristics**	**Frequency**	**Percent (%)**
Gender	Males	486	36.65
	Females	840	63.35
Educational attainment	Did not complete secondary	90	6.78
	Secondary diploma	128	9.66
	College diploma	133	10.03
	Bachelor or higher	975	73.53
Age	Under 18	24	1.81
	18 to 60	1,188	89.6
	Above 60	114	8.59
Community type	Commercial housing community	400	30.17
	Transitional succession community	309	23.30
	Traditional community	253	19.08
	Unit community	239	18.02
	Suburban community	125	9.43

### Measurements of the Constructs

A perceived social support questionnaire was used to measure the objective support, subjective support and support utilization perceived by residents in the closed management period of COVID-19 (48–50). The scale has 11 items. All items are measured on a 5-point Likert scale (1 = totally disagree; 5 = entirely agree), and the Cronbach's α was good (α = 0.931).

The social network questionnaire measured the scale, heterogeneity, and closeness of residents' relationship network and the degree of trust, reciprocity, and identity among residents, with a total of nine items (51–54). The Cronbach's α value of the scale in this study is 0.743, indicating good reliability and validity.

Epidemic prevention capability is measured with a 5-item scale that mainly includes four dimensions: prevention ability, rapid response ability, self-help ability, and recovery and reconstruction ability (30, 55). It explores residents' cognition and attitude toward the disease, coping ability and strategy effectiveness and runs through the three stages before, during and after the epidemic. In this study, the Cronbach's α was good (α = 0.811).

The psychological stress scale measured the residents' anxiety and panic about COVID-19, including the psychological stress response and negative emotions when coping with the epidemic situation during the closed management period, with a total of 18 items (56, 57). In this study, the Cronbach's α was good (α = 0.92).

With the transformation and development of Chinese society, the functions of urban communities have gradually diversified, and the differences in community attributes have become prominent (32). Social capital is heterogeneous in different types of communities, and the subjective well-being of residents in high social capital communities is generally higher (58), which in turn brings about differences in residents' emotions and health. At the same time, the more people trust other community members, the better their perceptions of their own health (59). The resources and service capabilities of a community are different, which magnifies or reduces residents' perceived social support, therefore affecting epidemic defense capabilities. Therefore, the type of community is selected as the moderating variable. There are five types: commercial housing communities, transitional succession communities, traditional communities, unit communities, and suburban communities (60).

### Statistical Analyses

The aim was to explore the epidemic defense capability of residents, community social network, perceived social support, and psychological stress during the epidemic. The mediating role of epidemic defense capability between community social support and psychological stress was revealed. We used SPSS 24.0 for the statistical analysis, including descriptive statistical analysis and correlation analysis. We used SmartPLS 3.0 for the structural equation model analysis, including model fitting effect testing, exploratory factor analysis, path analysis, and testing.

The confirmatory factor analysis, showed a high correlation among objective support, subjective support, and support utilization. This correlation is the premise of studying the higher-order dimension, so higher-order factor analysis is used to explore the correlation further. The specific steps are as follows: when there is a medium or high correlation between the first-order factors, higher-order factors can be extracted to explain the differences and relations between them, and the higher-order model can replace the lower-order model to improve the fit of the model to determine the best matching model (61). In this way, to simplify the model, we explored the composition of each dimension of the higher-order structure and enriched and developed the original theoretical framework.

## Results

### Common Method Bias and Reliability and Validity of the Scale

Although procedure control was carried out in the questionnaire design and survey implementation, there may still be common method bias because the same subject provided all variable data. Therefore, “Harman single-factor test” was used, and the results showed that the variance explained of the first principal component was 24.97%, which was <40% of the critical standard, so there was no common method deviation in this study.

**Figure 3:**
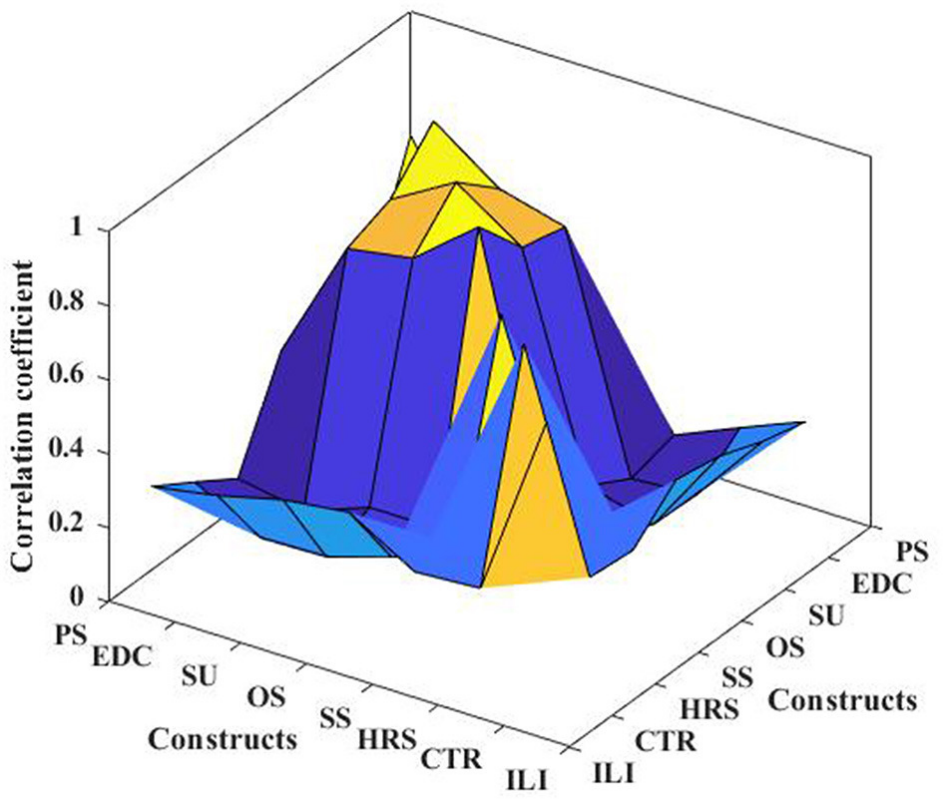
Correlation coefficient diagram.

**Figure 4:**
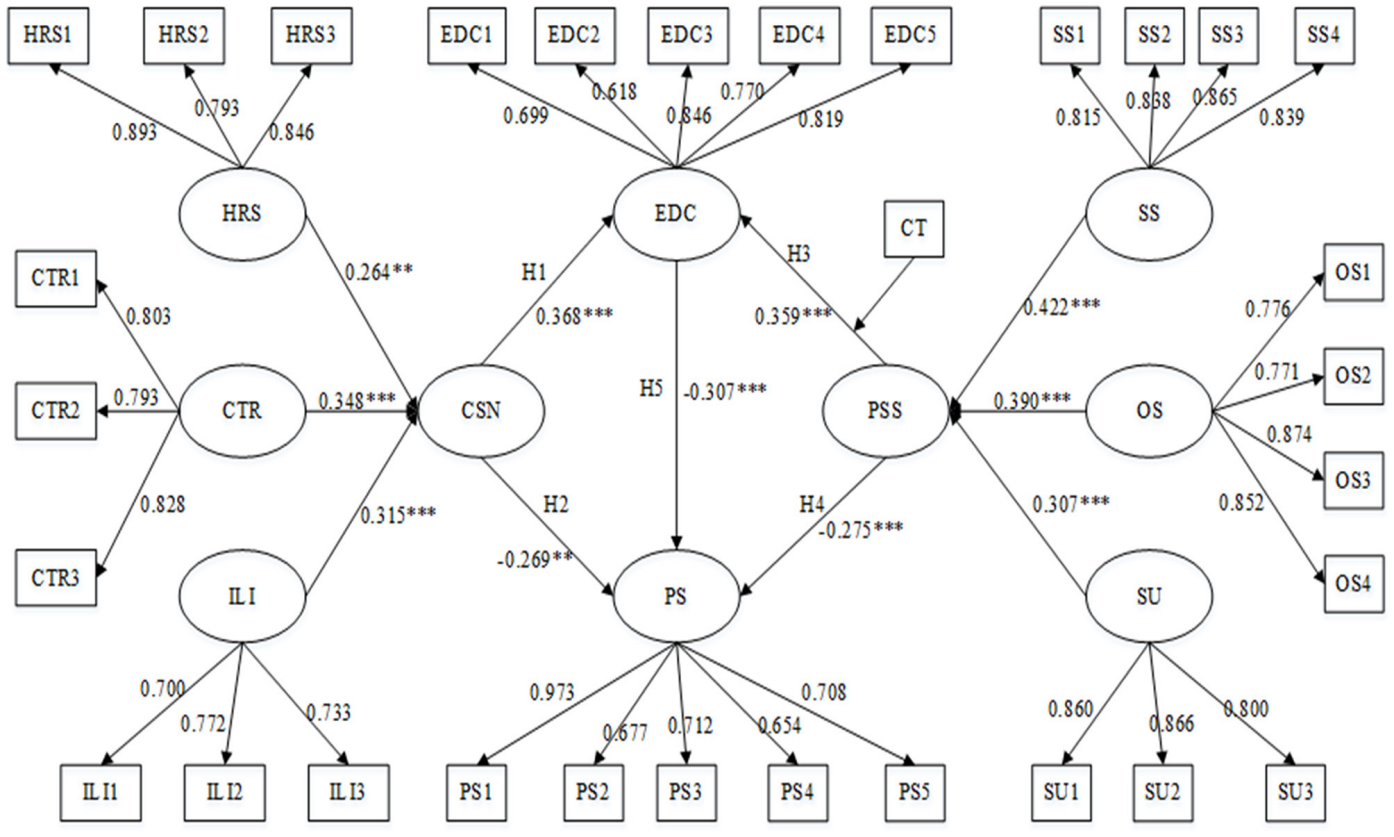
The results of the full model.

The KMO of all variables in the scale was larger than 0.7, indicating good validity and suitability for factor analysis. In Table 2, Cronbach's α > 7, CR > 0.7, AVE > 0.5, and all factor loading was >0.6, so the scale has high reliability, convergent validity and constructs validity. In addition, discriminant validity can be tested by the correlation coefficients between variables. Figure 3 shows that the load of the measurement item on the corresponding factor is greater than other factors, and the square root of the AVE of all variables on the diagonal is greater than the correlation coefficient between variables, so it meets the requirements of discriminant validity. Figure 4 shows the results of all model tests in the article.

**Table 2:** Results of EFA.

**Constructs**	**Items**	**Factor loading**	**Cronbach's α**	**CR**	**AVE**
Heterogeneity of Relationship Scale (HRS)	HRS1	0.893	0.743	0.778	0.659
	HRS2	0.793			
	HRS3	0.846			
Closeness of trust and reciprocity (CTR)	CTR1	0.803	0.743	0.852	0.658
	CTR2	0.793			
	CTR3	0.828			
Interactive learning identity (ILI)	ILI1	0.700	0.704	0.771	0.53
	ILI2	0.772			
	ILI3	0.733			
Community social network (CSN)	HRS		0.779	0.816	0.637
	CTR				
	ILI				
Subjective support (SS)	SS1	0.815	0.860	0.905	0.705
	SS2	0.838			
	SS3	0.865			
	SS4	0.839			
Objective support (OS)	OS1	0.776	0.836	0.890	0.670
	OS2	0.771			
	OS3	0.874			
	OS4	0.852			
Support utilization (SU)	SU1	0.860	0.795	0.880	0.710
	SU2	0.866			
	SU3	0.800			
Perceived social support (PSS)	SS		0.919	0.931	0.553
	OS				
	SU				
Epidemic defense capability (EDC)	EDC1	0.699	0.811	0.787	0.691
	EDC2	0.618			
	EDC3	0.846			
	EDC4	0.770			
	EDC5	0.819			
Psychological stress (PS)	PS1	0.973	0.820	0.757	0.633
	PS2	0.677			
	PS3	0.712			
	PS4	0.654			
	PS5	0.708			

### High-Order Structural Equation Model Testing

In the confirmatory factor analysis, it is found that there is a high correlation among the three dimensions of objective community support, subjective support, and support utilization. This interconnectedness is a prerequisite for studying higher-order dimensions. To describe their relationship and conduct a comprehensive analysis, this paper extracts two high-order factors, community social network and perceived social support, and builds a high-order factor model. From the path coefficient in Table 3, the closeness of trust and reciprocity has the greatest positive impact on community social network. This means that only close neighbors in the community can become social network nodes, and the tightness of the social network has the weakest relationship with the scale of the network.

**Table 3:** High order factor analysis.

**Paths**	**Estimate**	**Paths**	**Correlation coefficient**
SS → PSS	0.422^***^	SS—OS	0.699
OS → PSS	0.390^***^	SS—SU	0.669
SU → PSS	0.307^***^	OS—SU	0.716
HRS → CSN	0.264^**^	HRS—CTR	0.610
CTR → CSN	0.348^***^	HRS—ILI	0.705
ILI → CSN	0.315^***^	CTR—ILI	0.739

*** *P < 0.001*;

** *P < 0.01*.

In addition, subjective support has the greatest positive impact on perceived social support, indicating that the community provides psychological counseling to adjust pessimism can improve their perceived effectiveness of social support. A higher-order structure test showed differences in the degree of connection among the three types of support, and the relationship between support utilization and objective support was closer. This shows that the degree of utilization and acceptance of community support depends on the material assistance and services they receive; that is, what residents pay more attention to is objective support. In addition, the correlation between perceived social support and the first-order factors is strong, which indicates that the model conforms to the condition of higher-order factor analysis, and it is necessary to further explore the higher-order structure in theory.

### Hypothesis Testing of Structural Equation Model

The path analysis was conducted using the structural equation model. According to Henseler's model fitness criteria, we must select standardized residual mean root (SRMR) of the composite model when using the PLS algorithm. If the SRMR value is <0.1 or 0.08, then, the model has good fitness (62). The calculation results of this higher-order model show that SRMR = 0.069 <0.08. The goodness of fit of the model is good.

The path test results show that the community social network has a positive effect on the epidemic defense capability (β = 0.368, *P* = 0.000) and a negative effect on the psychological pressure (β = −0.296, *P* < 0.01). It can be seen from this that although institutional support from the government and other institutions is important, its main goal is to provide universal support, and there may often be “blind corner” that are difficult to cover. And other informal systems such as social networks can just play a role in supporting the formal complementary role of the system. Perceived social support can improve epidemic defense capability (β = 0.359, *P* = 0.000) and effectively alleviate psychological stress (β = −0.275, *P* = 0.000). The community maintains the mental health of the people and corrects the psychological deviations of the people promptly to block the spread of negative emotions in the community. Epidemic defense capability significantly impacted psychological stress (β = −0.307, *P* = 0.000). Therefore, H1 to H5 are supported (see Table 4).

**Table 4:** Verification table of path coefficient.

**Hypothesis**	**Path**	**Path coefficient**	**95% Confidence Intervals**	**Result**
H1	CSN → EDC	0.368^***^	[0.187, 0.394]	H1 is supported
H2	CSN → PS	−0.296^**^	[−0.156, −0.022]	H2 is supported
H3	PSS → EDC	0.359^***^	[0.233, 0.427]	H3 is supported
H4	PSS → PS	−0.275^***^	[−0.340, −0.156]	H4 is supported
H5	EDC → PS	−0.307^***^	[−0.384, −0.213]	H5 is supported

*** *P < 0.001*;

** *P < 0.01*.

### Mediating Effect Test Results

The Bootstrap method was used to analyze multiple mediating effects, which avoided the problem that the coefficient product test method might violate the distribution assumption. It also avoids inconsistent results due to different standard error formulas. Judgment is based on whether or not 0 is included in the 95% confidence interval. In this research, when using the method of repeated sampling, 5,000 bootstrap samples were randomly sampled from the original data (*N* = 1,326), and an approximate sampling distribution was generated (63).

In the higher-order factor model, the indirect effect value of perceived social support on psychological stress was 0.110 (*P* = 0.000, VAF = 28.6%). The indirect effect value of community social networks on psychological stress was 0.113 (*P* < 0.01, VAF = 27.6%). Epidemic defense capability plays a mediating role in both paths. Hypothesis H6 and H7 are confirmed (see Table 5).

**Table 5:** Bootstrap test of mediating effect.

**Independent variable**	**Mediating variable**	**Dependent variable**	**Direct effect**	**Indirect effect**	**Total effect**	**VAF (%)**
PSS	EDC	PS	−0.275^***^	−0.110^***^	−0.385^***^	28.6
CSN			−0.296^**^	−0.113^**^	−0.409^**^	27.6

*VAF is the Variance Accounted For, that is, the proportion of indirect effects to integrated effects. VAF value <20% means no mediation effect; 20% < VAF value <80% means partial mediation effect; VAF value >80% indicates complete mediation effect*.

*** *P < 0.001*;

** *P < 0.01*.

### Moderating Effect Test Results

Since perceived social support is a continuous variable and community type is a categorical variable, we should adopt the grouping regression method to test five groups of samples of different community types. As shown in Table 6, the *R*^2^ of the five regression equations was larger than 0.3, *P* < 0.01. The type of community has an obvious moderating effect on the path of perceiving social support and epidemic defense capability. Hypothesis H8 is confirmed.

**Table 6:** Moderating effect of community type.

**Community type**	**High Psychological stress**	**Low Psychological stress**	***R* ^2^**	**Adjusted *R*^2^**	**standard error**	***P-*value**
	**Percentage**	**Percentage**				
Commercial housing community	26.51	73.49	0.310	0.293	0.585	0.009
Suburban community	24.36	75.64	0.319	0.306	0.605	0.003
Traditional community	25.32	74.68	0.349	0.338	0.654	0.006
Unit community	18.79	81.21	0.315	0.311	0.549	0.000
Transitional succession community	30.57	69.43	0.373	0.368	0.593	0.000

## Discussion

The results of this article show that under the impact of major public health emergencies, community social networks can enhance defense capability and relieve psychological stress, mainly because efficient access to help can only be obtained through network members with close ties and high mutual trust. Interactive learning exists in the network every moment, the higher the identity of the members in the network, the higher the effective information flow. In the early stage of public health events, social network is one of the important channels for transmitting early warning and health information. At the same time, families' awareness of public health emergencies and their ability to integrate, absorb, and utilize resources is improved by learning tacit knowledge of social networks. Aldrich points out in his research that social capital generated by social networks can generate “community resilience” in times of crisis (59).

The structural equation model analysis results show that the perceived social support has a significant, positive impact on the epidemic prevention effect. This is consistent with Butel's findings that community residents benefit from collective efficacy (64). It mostly reduces the subject's evaluation of the severity of stressful events and provides problem-solving strategies to buffer the impact on health, thereby contributing to the mental health gains of disadvantaged groups. At the same time, its mediating effect is stronger than the community relationship network, indicating that the community's response to the people's appeals for epidemic prevention is more critical. The objective support of the community can meet the material needs of individuals, thereby enhancing defense capabilities, and subjective support can alleviate social problems, such as emotional alienation caused by isolation. In supporting utilization, promoting prevention and control policies and implementing measures to benefit the people are at the core of ensuring that community residents make full use of resources.

During the COVID-19 lockdown period, psychological stress was affected by both the public's social network and their perceived social support. Epidemic defense ability plays a mediating role in the relationship between social support and psychological stress, and the mediating effect accounts for 29.1% of the total effect. Our findings show that residents' attitudes (anxiety and confidence in fighting the epidemic) are significantly associated with the behavioral dimension of epidemic defense capability.

In this study, there were differences in the psychological stress of residents in different types of communities. A total of 18.79% of the residents in the unit community had high psychological stress reactions, while 30.57% of the individuals in the transitional succession community had high psychological stress reactions, which was significantly higher than that in other types of communities. The main reason is that it contains the characteristics of urban spatial morphology and includes the attributes of suburban communities. Most of them are in the management vacuum with strong population mobility and the greatest difficulty in prevention and control, resulting in a high degree of public anxiety. The traditional community neighborhood had a more heightened sense of security and identity, which can alleviate the psychological stress of the public.

The main significance of this study is as follows. Theoretically, this paper constructs a structural equation model from the individual and community levels to reveal the impact mechanism of psychological stress. In previous studies, both personal social networks and social support were regarded as social capital, and their unilateral effects were not studied (65, 66). This paper classifies community types in community-based research in terms of research content. There was no in-depth study of the differences brought about by them in the previous literature, or only a single community type was studied (16, 35).

## Conclusion

In conclusion, this study has key policy implications for preventing and controlling public health emergencies. First, in the government's institutionalized community prevention and control measures, there are often “blind corners” difficult to address. Other informal mechanisms attached to social networks can effectively eliminate residents' concerns about the development of the epidemic and closed control. Therefore, the government should carry out policy guidance and encourage residents to actively build a social network for epidemic prevention and use social capital. In addition, the social support perceived by community residents is closely related to the form of community organization, and the differences in individual information acquisition channels and risk-handling capabilities also result in a quite different focus on community support. Therefore, during the lockdown period, resources should be accurately allocated to help vulnerable communities with epidemic prevention, and the weak links of various community services should be improved in a targeted manner to alleviate psychological stress fundamentally.

## Limitations and Future Work

Several limitations of this study should be noted. In the data collection section, although invalid questionnaires have been excluded, the generalizability of the study is limited due to the demographic structure of different geographic regions. In some sites, highly educated populations were oversampled, yielding larger groups for comparisons. In the sampling process of the follow-up study, the representativeness and breadth of the sample should be enhanced by improving the survey method. At the same time, because psychological stress is self-reported, those with poorer self-regulation skills may experience more psychological stress during the lockdown. In the moderation effect test, since the community type is a categorical variable, the grouping regression method can only perform variance analysis, making the test results limited. Due to the sudden outbreak of COVID-19, there is very little research on the impact of individual and community levels on psychological stress, and we sought to make the theory more meaningful by combining different pieces of literature for a literature review. In future research, other moderating variables can be found by referring to relevant literature theories to clarify the influence mechanism of psychological stress further.

## Data Availability Statement

The datasets presented in this article are not readily available because, this dataset contains some private information that participants do not want to disclose. Requests to access the datasets should be directed to AJ, 15723243532@163.com.

## Ethics Statement

Ethical approval for this study and written informed consent from the participants of the study were not required in accordance with local legislation and national guidelines.

## Author Contributions

All authors contributed to the study conception and design. The first draft of the manuscript was written by XZ and AJ. All authors commented on previous version of the manuscript. All authors read and approved the final manuscript.

## Funding

The work was funded by Youth Fund for Humanities and Social Sciences Research of the Ministry of Education of the People's Republic of China (19YJCZH264), the Ethnic Research Project of the National Ethnic Affairs Commission of the people's Republic of China (2020-GMY-016), the Emergency Project of Social Science Investigation and Research on COVID-19 in the First-class discipline of Ethnology of Yunnan University (YNUXG-026), and the National Natural Science Foundation of China (71971093).

## Conflict of Interest

The authors declare that the research was conducted in the absence of any commercial or financial relationships that could be construed as a potential conflict of interest.

## Publisher's Note

All claims expressed in this article are solely those of the authors and do not necessarily represent those of their affiliated organizations, or those of the publisher, the editors and the reviewers. Any product that may be evaluated in this article, or claim that may be made by its manufacturer, is not guaranteed or endorsed by the publisher.
